# Dynamic stagnation: reasons for contraceptive non-use in context of fertility stall

**DOI:** 10.12688/gatesopenres.12990.1

**Published:** 2019-05-07

**Authors:** Apoorva Jadhav, Madeleine Short Fabic

**Affiliations:** 1 Office of Population and Reproductive Health, USAID, Arlington, VA, 22202, USA

**Keywords:** contraception, non-use, fertility, stall, transition, DHS

## Abstract

**Background:** While most countries have experienced reductions in the total fertility rate (TFR), there are countries where the fertility transition has not begun and others where the fertility transition has stalled with TFR still well above replacement level.  We use these contexts of “fertility stagnation” to explore reasons behind contraceptive non-use among women who desire to delay or avoid a future birth.  Specifically, we explore whether women’s reasons for not using contraception are stagnant as the population-level indicator, TFR, suggests or are more dynamic. This nuanced understanding can inform programmatic direction for countries especially in need.

**Methods:** Using Demographic and Health Survey (DHS) data from the two most recent surveys of ten study countries—five countries that have not yet experienced a fertility transition (“pre-transitional”) and five countries that began the fertility transition but stalled (“transitional”)—we explore trends in reasons for non-use.

**Results: **We find that that reasons for non-use are changing. Specifically, in pre-transition study countries, women are increasingly citing attitudes opposing contraception as reasons for non-use.  In transition countries, women are increasingly citing reasons related to service quality and method side effects. Perceived low fecundability is increasingly cited among both pre-transition and transition study countries. Geographical access and cost are not prominent frequently cited at either time point.

**Conclusions:** These findings highlight the need for prioritized programmatic attention aimed at: reducing opposition to contraception in pre-transitional countries; improving service quality and addressing issues of side-effects, both experienced and perceived, in transitional countries; and addressing fecundability perceptions in both types of country contexts.  We remind policy makers that even in contexts of fertility stagnation, underlying attitudes, norms, and barriers to contraceptive uptake are shifting.  Lack of change at the population-level can mask important changes at the individual-level that must be accounted for in program development and implementation.

## Introduction

While many countries have experienced reductions in the total fertility rate (TFR), there are some countries where the fertility transition ^1^ has not begun (“pre-transitional countries”, henceforth “PTC”) and others where the fertility transition has stalled with TFR still well above replacement level (“transitional countries”, henceforth “TC”). Fertility desires and infant mortality undoubtedly play a role in TFR changes or lack thereof ( Bongaarts, 2006; Westoff & Cross, 2006). Yet even in countries with high fertility desires, high infant mortality, and high TFR, there are still large proportions of women with unmet need for family planning (FP). Addressing unmet need for FP may have far reaching consequences, with the potential to reduce poverty and hunger and avert maternal and child deaths ( Cleland *et al*., 2006). Understanding the reasons behind why women are not using contraception when they have an explicit desire to delay or avoid a future birth is therefore of paramount importance, especially in countries in need of enhanced programmatic and policy attention (i.e., countries that are pre-transitional and countries with stalled fertility transitions). In this paper, we explore the reasons behind contraceptive non-use among women who desire to delay or avoid a future birth in countries with stalled or increasing TFR. 

Using Demographic and Health Survey (DHS) data from the two most recent surveys of 10 study countries—five PTCs and five TCs – our main objective is two-fold:

1) To determine whether reasons for contraceptive non-use are changing even when TFR is relatively unchanging; and
2) If reasons are changing, to identify the directionality of such changes to draw programmatic insight.

While it is important to note reasons for FP non-use among *all* women ( Chipeta *et al*., 2010; Fakeye & Babaniyi, 1989; Sahin & Sahin, 2003), it is of primary programmatic importance to focus on one subset of women – those women at risk of pregnancy who report a desire to delay or avoid a future birth. Our work builds on existing research on reasons for not using contraception among women with unmet need – women who want to stop or delay childbearing but are not using any method of contraception. Sedgh & Hussain’s (2014) seminal work on the reasons for contraceptive non-use among women with unmet need finds that increasing geographic access to family planning methods is insufficient to substantially reduce unmet need; addressing concerns of side effects and health risks is paramount. Similarly, in their examination of reasons for non-use across four countries, Choi *et al*. (2016) find that health concerns, including side effects, are major non-use factors women cited. In their study countries, perceived low fecundability is the most frequently cited reason. Letamo & Navaneetham (2015) find that opposition to use from partners is the main reason for non-use among women in Botswana, while Mekonnen & Worku (2011) find that commodity insecurity is a major reason in Ethiopia.

Our work contributes to and differs from this body of literature by a) focusing on countries experiencing fertility stall and b) analyzing the difference in women’s reasons for contraceptive non-use between two survey periods, a first for this topic. These data can inform programmatic focus for reducing unmet need. 

## Methods

Data are from Demographic and Health Surveys (DHS). The 10 datasets used for this study are available online from the DHS website: https://dhsprogram.com/data/available-datasets.cfm under the ‘Individual Recode’ subsection. Data can be accessed by applying through the DHS website. Please see their data access help page for information. DHS methodology is described in-depth elsewhere ( Short Fabic *et al*., 2012). In short, DHS is a nationally representative household survey that has been implemented in 90 countries with technical assistance provided by the DHS Program, supported since 1984 by the United States Agency for International Development. DHS provides data on population, health, and nutrition. All women between 15 and 49 years of age in sampled households are eligible for the women’s interview ^2^. Survey data pertaining to our study include total fertility rate, unmet need for FP, and women’s self-reported reasons for contraceptive non-use. 

We limit our analysis to those countries with at least two DHS conducted since 2000 with final datasets available as of March 2018. We further limit our analysis to those countries that have stalled or increasing TFR point estimates above replacement level (e.g. TFR > 2.1). We define stalled fertility based on Howse’s (2015) meta-analysis on the topic, which builds on work by Bongaarts (2008) and Shapiro & Gebreselassie (2008). Overall, there is agreement on broad notions of stalling fertility: a) fertility decline can only stall in countries where the fertility transition has already started. Thus, countries that are pre-transitional (e.g. TFR >/=5, per Bongaart’s categorization) are not considered as cases of stalling; b) fertility should have fallen in some way before stalling; c) countries where fertility is already close to replacement level are excluded. There is however, disagreement in the way these criteria are operationalized with regards to the distinction between pre-transitional and transitional, and the rate of fertility decline that constitutes a stall. We follow Howse’s (2015) broad grouping, and in cases where countries in his analyses are categorized based on older DHS surveys, we update the list based on the most recent data available. Our application of these criteria yielded a final sample of:

Five TCs: Dominican Republic, Egypt, Ghana, Indonesia, and Namibia; and
Five PTCs: Cameroon, Chad, Democratic Republic of Congo, Mozambique, and Niger.

Because we are especially interested in examining changes in reasons for contraceptive non-use, we limit our study population to women ages 15-49 who report themselves as ever having had sex, not currently pregnant, not currently using a contraceptive method, and who are clear about their desire to either limit or space future births (i.e., women who were filtered through the DHS questionnaire to answer the question on reasons for non-use, q709). Across our 10 study countries, there is a range of sample sizes of eligible women ( Table 1) On average at baseline, 1,072 women met our inclusion criteria in PTCs (range: 554 in Chad to 1,494 in Mozambique), while 1,604 women did so in TCs (range: 771 in Namibia to 3,263 in Indonesia). At endline, an average of 1,975 women met our inclusion criteria in PTCs (range: 1,227 in Niger to 2,754 in the DRC), while 1,497 did so in TCs (range: 497 in Dominican Republic to 2,989 in Indonesia). 

**Table 1.** Sample characteristics – total fertility rate (TFR) and sample sizes for women (15–49) in each survey and eligible for question on reasons for not using contraception at baseline and endline by country.

	Baseline				Endline				Inter-Survey		
Country	Survey year	Survey sample	Eligible women for q709	TFR (SE)	Survey year	Survey sample	Eligible women for q709	TFR (SE)	Years	TFR Change	Avg. annual change *
(Chad)	2004	6,085	554	6.35 (0.15)	2014	17,719	1,937	6.45 (0.09)	10	0.098	0.010
(DRC)	2007	9,995	1,227	6.29 (0.18)	2013	18,827	2,754	6.57 (0.12)	6	0.281	0.047
(Mozambique)	2003	12,418	1,494	5.53 (0.10)	2011	13,745	2,014	5.92 (0.10)	8	0.389 *	0.049
(Niger)	2006	9,223	1,041	7.09 (0.11)	2012	11,160	1,227	7.64 (0.10)	6	0.542 *	0.090
(Cameroon)	2004	10,656	1,043	4.97 (0.10)	2011	15,426	1,943	5.09 (0.10)	7	0.119	0.017
Dominican Republic	2007	27,195	1,547	2.43 (0.05)	2013	9,372	497	2.48 (0.07)	6	0.045	0.008
Egypt	2008	16,527	1,566	3.02 (0.04)	2014	21,762	1,972	3.47 (0.04)	6	0.444 *	0.074
Ghana	2008	4,916	872	4.03 (0.13)	2014	9,396	1,367	4.19 (0.12)	6	0.167	0.028
Indonesia	2007	32,895	3,263	2.59 (0.04)	2012	45,607	2,989	2.60 (0.04)	5	0.007	0.001
Namibia	2006	9,804	771	3.57 (0.09)	2013	9,176	658	3.65 (0.09)	7	0.08	0.011
**Average**				4.6				4.8	6.7	0.219	0.033
**PTC**				6.1				6.3	7.4	0.288	0.039
**TC**				3.1				3.3	6.0	0.15	0.025

Note: Countries marked in parentheses are those in “pre-transition” as per Bongaarts’ definition. Countries marked with * are where the TFR change is statistically significant at the 0.05 level * Not precisely an annual change since TFR calculated in each DHS Survey is a 3-year average from the time of interview. PTC – pre-transitional countries, TC – transitional countries

Our main variable of interest is women’s self-reported reason(s) for not using any method of contraception. Eligible women are asked an open-ended question (q709), “Can you tell me why you are not using a method to prevent pregnancy?” Once she provides a reason, the respondent is probed by the interviewer, who asks if there is any other reason besides the one stated. Thus, respondents are able to provide multiple reasons for not using contraception, though the majority reports only one reason. The interviewer then codes these responses into 23 pre-structured categories. Over time, these pre-structured categories have expanded to more precisely capture the range of responses women provide. Additionally, these pre-structured categories are sometimes modified based on country context. 

To overcome these survey questionnaire differences, we further group responses into seven categories reflecting elements of family planning access that align with *Choi et al*.’s framework on measuring access to family planning ( Choi *et al*., 2016). These categories are:

**Table:** 

*Cognitive*	(knows no method, knows no source);
*Psychosocial*	(respondent opposed, husband/ partner opposed, religious prohibition, fatalistic/up to God);
*Geographic*	(lack of access/too far);
*Cognitive and Quality*	(interferes with body’s normal processes, inconvenient to use, side effects/health concerns, preferred method not available ^3^, no method available ^2^);
*Affordability*	(costs too much ^4^);
*Perceived low* *fecundability*	(not having sex ^5^, infrequent sex, menopausal/hysterectomy, cannot get pregnant/difficult to get pregnant, not menstruated since last birth ^6^, breastfeeding ^7^, too old ^8^, husband away ^7^);
*Other*	(other, don’t know).

Our grouping of responses by access element differs slightly from the Choi *et al.* framework. Specifically, we group the access element of *quality* in Choi *et al.*’s framework (“preferred method not available” and “no method available”) into the framework’s “ *cognitive and quality*” access element. We make this change because most study country baseline surveys did not include the two *quality* codes. Moreover, we conducted separate sensitivity analyses excluding and including these two responses in the “ *cognitive and quality*” category, data from which yielded similar conclusions. With regard to country-specific modifications to q709, we found that the baseline survey in Indonesia contained two extra responses, “too old” and “husband away,” which we added to the *perceived low fecundability* access category.

We present largely descriptive results, detailing the change in reasons behind contraceptive non-use at two different time points. Analyses are adjusted for sample design in each survey using appropriate survey weights, stratification, and primary sampling unit variables ( Croft *et al*., 2018). To assess statistically significant changes between surveys, we use two-tailed tests applying survey weights. P-values less than 0.05 are considered statistically significant. We use STATA 14.2 for all analyses.

## Results and discussion

### Fertility context

On average across all study countries, TFR change is 0.2 in the inter-survey period (see Table 1). Looking at TFR by transition status, average TFR at baseline is 6.1 in PTCs and 3.1 in TCs. At endline, average TFR is slightly higher in both sets of countries, 6.3 in PTCs and 3.3 in TCs. Meanwhile, mean ideal number of children varies little between baseline and endline in both PTCs and TCs. In PTCs, mean ideal number of children is, on average, 7.0 at baseline and 6.8 at endline. In TCs, mean ideal number of children is on average 3.2 at both baseline and endline (data not shown). 

### Family planning context: Do unmet need and contraceptive prevalence change in countries experiencing fertility stagnation?

Turning our attention to changes in unmet need over time across study countries, we find that unmet need at baseline is high, averaging 20% across study countries ( Table 2a). At endline, average unmet need across study countries is unchanged at 20%. Unsurprisingly, unmet need is highest in PTCs (average 23% at endline) in contrast with transition countries (average 16% at endline). We also find that the directionality of changes in unmet need varies based on transition status. In all five PTCs, unmet need remained constant or increased over time (average increase of 2 percentage points), whereas in all five TCs, unmet need either remained constant or decreased over time (average decrease of 2 percentage points). Overall, we find that fertility stalls and increases are met with high and persistent unmet need. 

**Table 2a.** Change in unmet need between survey years by country.

	Unmet need			
Country	Baseline (%)	Follow-up (%)	Difference (% point)	p-value
(Chad)	20.6	22.9	2.3 *	0.012
(Democratic Republic of Congo)	26.9	27.7	0.8	0.201
(Mozambique)	18.9	23.9	5.0 *	0.000
(Niger)	16.1	16.0	-0.1	0.605
(Cameroon)	20.5	23.5	3.0 *	0.001
Dominican Republic	11.1	10.8	-0.3	0.793
Egypt	11.6	12.6	1.0 *	0.011
Ghana	35.7	29.9	-5.8 *	0.002
Indonesia	13.1	11.4	-1.7 *	0.000
Namibia	20.7	17.5	-3.2	0.201
**Average**	19.5	19.6	0.1	
**Pre-transition countries**	20.6	22.8	2.2	
**Transition countries**	18.4	16.4	-2.0	

Note: Countries marked with * are where the difference is statistically significant at the 0.05 level. Countries marked in parentheses are those in “pre-transition” as per Bongaarts’ definition.

With regard to contraceptive prevalence (CPR), we find that—as expected—CPR differs dramatically between the PTC and TC contexts and is much lower in PTCs ( Table 2b). Over the inter-survey period, PTCs experienced an average decrease in CPR of 2 percentage points (17% at baseline to 15% at endline) whereas TCs experienced on average no change in CPR (55% at baseline and endline). 

**Table 2b.** Change in contraceptive prevalence (any method, in-union women) between survey years by country.

	CPR			
Country	Baseline (%)	Follow-up (%)	Difference (% point)	p-value
(Chad)	11.1	5.7	-5.4 *	0.000
(Democratic Republic of Congo)	20.6	20.4	-0.2	0.201
(Mozambique)	16.5	11.6	-4.9 *	0.000
(Niger)	11.2	13.9	2.7 *	0.003
(Cameroon)	26.0	23.4	-2.6 *	0.013
Dominican Republic	72.9	71.9	-1.0	0.341
Egypt	60.3	58.5	-1.8 *	0.014
Ghana	23.5	26.7	3.2 *	0.028
Indonesia	61.4	61.9	0.5	0.453
Namibia	55.1	56.1	1.0	0.493
**Average**	35.9	35.0	-0.8	
**Pre-transition countries**	17.1	15.0	-2.1	
**Transition countries**	54.6	55.0	0.4	

Note: Countries marked with * are where the difference is statistically significant at the 0.05 level. Countries marked in parentheses are those in “pre-transition” as per Bongaarts’ definition.

In summation, PTCs on average witnessed increases in unmet need and decreases in CPR, while TCs on average witnessed decreases in unmet need and no change in CPR. 

### Have reasons for contraceptive non-use changed as TFR has stalled or increased?

With regard to reasons for contraceptive non-use, we find most study countries have witnessed changes over time in the predominant reasons women cite, regardless of transition status. These changes are statistically significant for all countries except the DRC. We also observe divergent trends in reasons for non-use by transition status, though for a handful of reasons, trends are virtually universal across all study countries, as described further herein. 

First, we examine reasons for non-use at the country-level ( Table 3a for PTCs and Table 3b for TCs). We find that among all study countries, Niger witnessed the biggest changes between survey rounds, followed by Chad. Both countries saw large declines in reasons cited related *cognitive* access and large increases in reasons cited pertaining to *perceived low fecundability*. Meanwhile, Egypt and Namibia witnessed the smallest changes in reasons for non-use between survey cycles with Egypt seeing slight increases in *cognitive and quality* reasons and slight decreases in reasons related to *perceived low fecundability*. Namibia also saw slight decreases in reasons related to *perceived low fecundability* as well as in *cognitive and quality* reasons, coupled with slight increases in *affordability*-related reasons. Niger witnessed the biggest changes in reasons for non-use among all study countries with massive changes across a host of reasons for non-use. While knowledge of contraceptive methods and services became less of a barrier to FP use in Niger, opposition became a bigger barrier, indicating that while *cognitive* access is improving, *psychosocial* access may be worsening. In Ghana, service-related barriers to use are becoming more prevalent, with *cognitive and quality* access, *geographic* access, and *affordability*-related reasons for non-use increasingly representing a larger proportion of reasons for non-use. 

**Table 3a.** Difference (% point) in reasons for not using contraception between baseline and endline by country for women 15–49 with unmet need in Pre-Transitional Countries.

*Pre-transitional Countries*		Related element of access							*p-value*
		Cognitive	Psycho- social	Cognitive & Quality	Geographic	Affordability	Perceived low fecundability	Other	
**Chad**	Baseline	22.4	27.5	16.4	0.3	2.8	19.7	11	
	Endline	14.4	21.6	8.6	0.5	1.8	43.8	9.3	
	Change	-7.9	-5.9	-7.8	0.3	-0.9	24.1	-1.7	0.000 *
**DRC**	Baseline	15.9	17	21.6	1.1	5.1	30.8	8.6	
	Endline	12.6	19.3	21.5	1.4	2.4	35.4	7.4	
	Change	-3.3	2.3	-0.1	0.3	-2.7	4.6	-1.2	*0.085*
**Mozambique**	Baseline	7.2	12.7	11.7	6.6	2.6	47.7	11.6	
	Endline	1.6	26.7	8.8	4.5	6	47.4	5.1	
	Change	-5.6	14	-2.9	-2.1	3.4	-0.3	-6.6	0.000 *
**Niger**	Baseline	19.6	25	19.2	3.8	3.9	17	11.5	
	Endline	5.8	35.6	12	4.2	1	35.7	5.7	
	Change	-13.8	10.5	-7.1	0.4	-3	18.7	-5.8	0.000 *
**Cameroon**	Baseline	15.8	10.5	11.5	0.4	3.3	39.7	18.8	
	Endline	12.6	13.2	20.6	0.7	8.1	32.1	12.7	
	Change	-3.2	2.7	9.1	0.3	4.8	-7.6	-6.1	0.000 *

Note: Countries marked with * are where the difference is statistically significant at the 0.05 level.

**Table 3b.** Difference (% point) in reasons for not using contraception between baseline and endline by country for women 15–49 with unmet need in Transitional Countries.

*Transitional* *Countries*		Related element of access							*p-value*
		Cognitive	Psycho-social	Cognitive & Quality	Geographic	Affordability	Perceived low fecundability	Other	
**Dominican** **Republic**	Baseline	0.9	16	26	0.5	1	41.1	14.5	
	Endline	0.6	17.8	32.5	0.7	0	37.3	11	
	Change	-0.3	1.8	6.5	0.2	-1	-3.8	-3.4	0.000 *
**Egypt**	Baseline	0.1	11.6	33.3	0.1	0.6	51.3	2.9	
	Endline	0.2	12.7	36.6	1.1	0.9	47	1.7	
	Change	0.1	1	3.2	1	0.3	-4.4	-1.2	0.002 *
**Ghana**	Baseline	5.2	15.8	38.5	0.5	3.4	29.6	7	
	Endline	1.1	15.2	43.9	4.6	5.1	24.5	5.7	
	Change	-4.1	-0.7	5.4	4.1	1.7	-5.1	-1.3	0.000 *
**Indonesia**	Baseline	0.8	3.7	31.1	0.5	5	39.4	19.5	
	Endline	0.8	3.6	30.1	0.2	2.9	24.5	37.9	
	Change	0	-0.2	-1.1	-0.3	-2.1	-14.9	18.4	0.000 *
**Namibia**	Baseline	3.8	10.8	25.6	3.3	5.3	27	24.2	
	Endline	1.2	10.8	22.5	4.7	8.9	25.7	26.1	
	Change	-2.6	0	-3.1	1.4	3.6	-1.3	1.9	0.000 *

Note: Countries marked with * are where the difference is statistically significant at the 0.05 level.

### Do shifts in reasons for non-use differ by transitional status?

**Figure 1.**
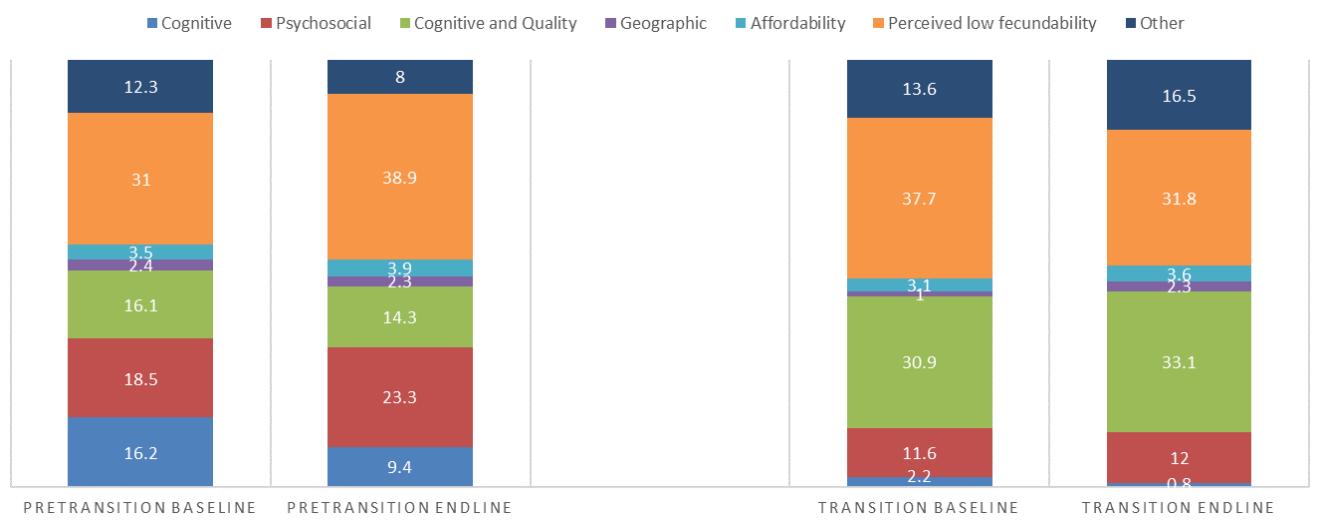
Elements of access between baseline and endline by transition status, women 15-49 in all survey countries.

It is more useful to look at overall differences by transition status, recognizing that illuminating the categories of difference can be programmatically relevant. In doing so, we find striking differences ( Figure 1). Among PTCs, the biggest shifts over time were witnessed in reasons related to *cognitive* access barriers, *psychosocial* access barriers, and *perceived low fecundability*. *Cognitive* reasons for non-use dramatically declined. Conversely, *psychosocial* reasons for non-use increased substantially, as did reasons related to *perceived low fecundability*. Among TCs, the shifts in reasons for non-use were less dramatic. The biggest change was a decrease in reasons related to *perceived low fecundability*. More minor changes witnessed included increases in reasons related to *cognitive and quality* access and reasons classified as “ *other*”. 

There are enormous differences in reasons for non-use between PTCs and TCs ( Figure 1). To elaborate, at baseline, *cognitive* access barriers are on average 8x higher in pre-transition countries than in transition countries. This gap between pre-transition and transition countries holds true at endline. Similarly, *psychosocial* barriers are much higher at both baseline and endline among PTCs as compared with TCs. For TCs, reasons for non-use related to *cognitive and quality* access were much more frequently reported at both baseline and endline as compared with PTCs. Finally, one difference between PTCs and TCs reversed course between baseline and endline. That is, *perceived low fecundability* reasons for non-use were higher among TCs at baseline and higher among PTCs at endline. These data indicate that common reasons for non-use among countries with stalled or increasing TFR vary by transitional status, as would be expected. Furthermore, even when TFR is stagnant or increasing, reasons for non-use are changing. 

Finally, there are several trends in a subset of reasons for non-use that span nearly all study countries, regardless of transitional status. First, *cognitive* reasons for non-use are declining across study countries. This indicates that a core foundation of behavior change—knowledge—is becoming less of an impediment to FP access in countries with stagnant or increasing TFR. Second, *psychosocial* access barriers are stagnant or increasing across all study countries but for Chad. Even in Chad, *psychosocial* barriers still represent nearly 22% of all reported reasons for non-use at endline. Indeed, *psychosocial* barriers to contraceptive use are high at both baseline (average 15%) and endline (average 18%) across study countries. As these data show, another key element of behavior change—attitudes—has been and remains a major challenge for expanding contraceptive use among women who wish to delay or limit their next birth in counties with stalled or increasing TFR. 

Turning to reasons for non-use related to *geographic* accessibility, the data show that geographic barriers to access are not frequently cited at either baseline or endline in study countries. Interestingly, while most countries saw little to no change in geographic reasons for non-use over time, Ghana witnessed an increase of four percentage points. *Affordability*-related reasons for contraceptive non-use were also low across study countries at baseline and remained low at endline. The biggest shift was witnessed in Cameroon, which saw an increase in affordability-related reasons of nearly five percentage points. These data show that overall issues of *affordability* and *geographic* accessibility are not frequently cited by women living in countries with stalled or increasing TFR, indicating that other areas influencing family planning access may be of higher programmatic priority in these country contexts. 

Finally, the reasons cited under “ *other*”, which include “don’t know” also are decreasing across all study countries except Indonesia, which could be a signal that women are increasingly able to identify and describe their reasons for non-use. This finding could also be a reflection of changes in DHS core questionnaire answer codes, which have expanded over time to cover additional types of reasons, thereby reducing the need for “ *other.*”

## Conclusions

There are four broad trends emerging from our work. First, despite there being stalls or increases in TFR across study countries, unmet need actually increases or decreases depending on transition status with PTCs experiencing slight increases and TCs experiencing slight decreases. Second, while contraceptive knowledge is improving across all countries, the *cognitive* access gap remains very high between PTCs and TCs and must continue to be addressed in pre-transitional contexts. Third, *geographic* access and *affordability* are not prominent reasons women in our study countries cite for not using contraception, signaling other elements of access must first be addressed in order to reduce unmet need. Finally, our analysis indicates that there are certain access elements that require immediate programmatic attention: *Psychosocial* access and *perceived low fecundability* are the main access barriers to address in PTCs while *cognitive and quality* access barriers are paramount in TCs. We use this opportunity to discuss several programmatic actions required to address these barriers to family planning access and use.

To overcome *psychosocial* barriers, it is critical to continue investing in social and behavior change, especially as related to men’s involvement and engagement. Male engagement in family planning ( Abdur-Rahman *et al*., 2018), acknowledging men as actors rather than mere bystanders ( Hardee *et al*., 2017), dispelling common misconceptions about family planning ( Kabagenyi *et al*., 2014; Muanda *et al*., 2016; Withers *et al*., 2010) and training providers on new methods like no-scalpel vasectomy and other male-friendly family planning services as under the successful *Permanent Smile Campaign* project in Ghana ( Subramanian *et al*., 2010) are critical to addressing high and persistent unmet need in countries experiencing fertility stagnation.

While some *cognitive and quality* access barriers require social and behavior change interventions—like addressing widespread myths and misperceptions ( Gueye *et al*., 2015)—many others require improvements in service delivery environment, especially as related to effective counseling and method choice. For example, Machiyama & Cleland (2014) find that Ghanaian women – particularly urban educated women – may have a resistance to using hormonal contraception due to past experience with side effects, leading to reliance on traditional and less effective contraceptive methods instead. Another recent study in Bangladesh found that many women discontinued contraceptive use due to the negative impact contraceptive side effects had on their participation in various life activities, especially the impact of irregular bleeding on religious life ( Jain *et al*., 2017). To address *cognitive and quality* issues impacting access, enhanced programmatic action is especially needed to: a) expand method choice among a wide variety of contraceptive methods and support method switching, recognizing that side effects are real and represent real problems; and b) enhance counseling and information exchange to with women and men on how to use methods and what to expect while using them, to dispel myths, and explain and address side effects. 

*Perceived low fecundability* is a major reason for non-use cited by women across all ten study countries. High levels of perceived low fecundability may reflect a low awareness of one’s risk of pregnancy. This is particularly important for postpartum women who do not often have correct knowledge about return to fertility. Indeed, unmet need is as high as 65 percent among postpartum women ( Gaffield *et al*., 2014; Pasha *et al*., 2015). Evidence from 17 countries shows that the return of menstruation is the only marker associated with increased modern contraceptive use among postpartum women ( Borda *et al*., 2010), which leaves a window of time that women may be susceptible to an unintended pregnancy. 

Given that the DHS continues to be the main source of information on contraceptive non-use in developing countries, it is important then to ensure that household surveys like the DHS are able to better capture the reasons women are not using contraception. As Staveteig & Juan (2018) suggest, the DHS women’s questionnaire could contain better probes to capture a) whether women are underreporting traditional method use and b) sources of their information about perceived side effects – own, those of their friends, etc.

Our analyses reveal that indeed, lack of change at the population-level can mask important changes at the individual level. We remind programmers and policy makers that even in context of TFR stagnation, underlying attitudes, norms, and barriers to contraceptive uptake are shifting and programmatic action must shift accordingly.

## Data availability

### Source data

Data used in this study is available online from the Demographic and Health Survey (DHS) website. The relevant datasets, available under the ‘Individual Recode’ subsection, are:

- Cameroon (2004, 2011)
- Chad (2004, 2014)
- Democratic Republic of Congo (2007, 2013)
- Dominican Republic (2007, 2013)
- Egypt (2008, 2014)
- Ghana (2008, 2014)
- Indonesia (2007, 2012)
- Mozambique (2003, 2011)
- Namibia (2006, 2013)
- Niger (2006, 2012)

Data can be accessed by applying through the DHS website. Please see their data access help page for information.

## Footnotes

^1^Defined and described in detail in the “Data and Methods” section.

^2^Only ever-married women are interviewed in a subset of countries in Asia, North Africa and Middle East due to cultural sensitivities.

^3^These questions were only included in the endline surveys for all countries due to questionnaire revisions between survey phases.

^4^Could refer to transportation cost, which reflects both geographic accessibility and affordability

^5^Accuracy of the perception cannot be assessed.

^6^If women’s perception is based on incorrect knowledge of return of postpartum fertility, response may reflect barriers in cognitive accessibility.

^7^Response can also be related to service quality if women implied health concerns related to breastfeeding and contraception that were not properly addressed.

^8^Only in the Indonesian baseline survey
