# Supplementary material for: Dynamic stagnation: reasons for contraceptive non-use in context of fertility stall
Source: Gates Open Res. 2019 May 7;3:1458. [Version 1] doi: 10.12688/gatesopenres.12990.1 (PMC10547112; doi:10.12688/gatesopenres.12990.1)
Supplement: Peer review history

# Reviewer report: Reviewer response for version 1

**DOI:** 10.21956/gatesopenres.14097.r28689

**Published:** 2020-04-24

**Authors:** Kannan Navaneetham

**Affiliations:** 1 Department of Population Studies, University of Botswana, Gaborone, Botswana

The main objective of this paper is to examine the reasons for non-use of contraception among women who desire to delay or avoid births in the context of stalling fertility using data from Demographic Health Surveys (DHS) of 10 countries. This paper also tried to draw inferences about how the reasons for non-use of contraception vary between pre-transitional countries (PTC) and transitional countries (TC). Understanding the dynamics of unmet need for contraception and the reasons for not using it is a programmatic importance and have policy implications for the implementation of family planning programme in developing countries. However, I have a major problem with this paper with respect to analytical content, data analysis and interpretation.

General comments:

- The analysis could have at the disaggregated level of women who desire to delay and avoid births, that is separate analysis for reasons for non-use of contraception for spacing and for limiting. This is because the reasons for non-use of contraception would vary depending whether the women want to delay or avoid births. If women want to delay the births, the reasons for non-use of contraception mostly pertaining to spacing methods and if they want to avoid births their reasons could be pertaining to terminal methods. The analysis done without disaggregating this way may be problematic and hide important information.
- Various demographic and socioeconomic factors are contributing for a woman to delay the births or to avoid births. The most important demographic factors are age and parity. The age and parity distribution are different between the PTC and TC, without controlling these factors, may tend to have erroneous conclusion. My question is whether we can make meaningful comparison on the reasons for non-use, if those countries are different with respect to age structure, parity distribution, distribution of family planning method use, variation of cultural beliefs and norms etc. Literature shows that most unmet need contribute from women who wish to postpone the next birth in Sub-Saharan African countries, whereas in Asia and Latin America unmet need results primarily from those who want to terminate childbearing. These differences would have implications on interpreting the results of reasons for non-use of contraception.

Now I outline below the specific comments in each of sub-heading of the paper.

**Introduction:**

 There are numerous studies in developing countries either stand alone or in a comparative perspective which addressed the reasons for non-use of contraception among women with unmet need. The most important one is by Sedgh & Hussain (2014) ^1^, as this paper also cited. I am unable to figure out from the introduction, what is the motivation for this paper and how it is deviating from the earlier studies and findings, for example from study by Sedgh & Hussain (2014) ^1^. Previous studies have also reported how the reasons vary between high fertility and low fertility countries. However, it is unclear, why the authors want to compare between countries with stalling fertility of high and low? What are the existing knowledge gaps? What the author is hypothesizing or why author expects that reasons could be different between these two set of countries. How the paper is going to add value into the existing literature. What is the critical research problem here in the context of stalling fertility decline? The introduction needs proper motivation and contextualization of research problem.

**Methods**

 The study selected 10 countries and among them five are from PTC and another five from TC. The criteria for selecting these countries are:

1. with at least two DHS conducted since 2000 with final datasets available as of March 2018.
2. limit analysis to those countries that have stalled or increasing TFR point estimates above replacement level (e.g. TFR > 2.1).

The selection of countries using above criteria seems to be arbitrary. Regarding criteria (a), the paper did not give any justification or rational for the cut-off year 2000 for choosing the countries. Why not before 2000? What is the significance of this year?  I suggest that the authors should indicate the justification for this.

 The countries selected are:

 Five TCs: Dominican Republic, Egypt, Ghana, Indonesia, and Namibia; and

 Five PTCs: Cameroon, Chad, Democratic Republic of Congo, Mozambique, and Niger

 The paper does not give rational and procedure for choosing these countries in PTC and TC. To be specific, how those countries were selected? In total, how many countries the fertility was stalling? From those countries, how you finally arrived to select these countries? Further, in both groups, all countries are from Africa except Indonesia and Dominican Republic. Why Indonesia and Dominican Republic were chosen for the analysis? Are there any specific reasons? Are those countries being homogeneous or heterogeneous in certain characteristics (for example supply side factor, similar strategy for the implementation of family planning programme, cultural factor, religious factors, or any other factors). If so, how and why. I would appreciate specifying rational for the selection of the countries within the group, apart from fertility stall.

 The inclusion criteria used for the study population is: “women ages 15-49 who report themselves as ever having had sex, not currently pregnant, not currently using a contraceptive method, and who are clear about their desire to either limit or space future births”. However, this criterion is different from algorithm used for estimating the unmet need. Not clear, all women or only ever married women were selected. The note no. 2 in the paper says that *only ever-married women are interviewed in a subset of countries in Asia, North Africa, and Middle East due to cultural sensitivities*. Need some clarification. Suppose all women were selected, combining unmarried women and married women, and understanding reasons for non-use of contraception may be problematic. In Africa, one-fourth of the unmet need women are from unmarried women and the reasons for non-use expected to be different.

 Women ever having had sex may not be currently sexually active. The algorithm used for estimating unmet need incudes currently pregnant and amenorrheic women if they say that their current or last pregnancy was unintended. But why you have excluded currently pregnant women. What about infecund women? Are they excluded? Need more clarification on the filtering of the study population.

 The main variable is women’s self-reported reason(s) for not using any method of contraception. Eligible women are asked an open-ended question (q709), “Can you tell me why you are not using a method to prevent pregnancy?” The major limitation of this question is that it is a self-reported and very subjective in nature. The information from this question should be used cautiously. Studies have shown that women are reluctant to identify their own ignorance as a reason for non-use as it may doubt their self-respect (Cleland, Harbison, and Shah, 2014 ^2^). The responses from this question is not consistent when compared with other information or other questions. For discussion on this review, refer to Cleland, Harbison, and Shah (2014) ^2^. The authors need to bring those discussions and include the limitation of using these subjective responses for interpreting the results.

 Baseline and End line years are different for different countries and intervals (ranging from 5 to 10 years) are different between countries. Are the results comparable among countries when different periods were used for studying change? The authors should justify how the results are not affected by these variations in the duration.

**Results and Discussion**

 It is interesting to note that the mean ideal number of children has not changed much during the base year and end year in both PTC (an average of 6.9 children during the period) and TC (an average of 3.2 children). Consequently, the fertility has not declined, and it was stalling in these countries. This implies that the main factor for stalling fertility in these countries was due to constant desired family size driven by poor female education, greater benefits of having more children and lack of modernization. In this context, these factors are more important for considering policy relevance for socioeconomic development whereas unmet need and their reasons for not using contraception could be less important for discussion and deriving policy priorities for family planning. The paper needs through discussion on these aspects.

 I suggest that results section should starts with the selected characteristics of the study population and discussion on how these characteristics vary between countries. A table can be given. Because these differences will have implications on the interpreting the results of reasons for non-use of contraception.

 Table 2a gives the unmet need for base year and end year and changes over the period. The definition and measurement of unmet need in the DHS surveys have changed over time (Bradley et a., 2012 ^3^). Whether an adjustment on the estimation of unmet need was made for comparing between countries and over the time period? Need clarification on this. It is interesting to note that Ghana, the TC with the total fertility rate of about 4 children per woman and contraceptive prevalence rate of 25%, has higher unmet need compared to all the PTC with the TFR of more than 6 children. Similarly, Niger has low unmet need but high fertility. Probably, lower fertility in Ghana could be attributed due to abortion prevalence in the country compared to PTC. It could be that the desired family size in Niger is larger and therefore policies related to reducing unmet need may not be central importance than improvement in socioeconomic development in the country. Need more discussion on these nuances in a comparative perspective.

 The table revealed that there is a variation on the reasons for non-use within PTC and TC. For example, psychosocial barrier is higher in all the PTC countries except Cameroon where it is closer to TC countries. The author has not brought this type of differences within the group of countries and explanation of those variation.

 The authors should be cautious in interpreting the table. I quote from the paper “In Ghana, service-related barriers to use are becoming more prevalent, with *cognitive and quality access*, *geographic access*, and *affordability*-related reasons for non-use increasingly representing a larger proportion of reasons for non-use”. I suggest the authors to check the table and interpret correctly. According to the table 3a, the dominant reasons in Ghana are *cognitive and quality access, perceived low fecundability and psychosocial*.

 In some of the countries, majority of the women reported, particularly Indonesia and Namibia, as *other reasons, which include don’t know* (about one-fourth). Unfortunately, we do not know what those other reasons are. It would be also interesting to see who those women are and what are their characteristics.

 The findings are not new, and conclusion is vague and some of the explanations are superfluous not supported by the evidence.

 In sum, the paper is weak both analytically and technically. This paper needs considerable revision. I hope my comments would be helpful to the authors to improve the quality of the paper.

Is the work clearly and accurately presented and does it cite the current literature?

Partly

If applicable, is the statistical analysis and its interpretation appropriate?

Partly

Are all the source data underlying the results available to ensure full reproducibility?

Yes

Is the study design appropriate and is the work technically sound?

Partly

Are the conclusions drawn adequately supported by the results?

Partly

Are sufficient details of methods and analysis provided to allow replication by others?

Yes

Reviewer Expertise:

Demography, Health and mortality, Population and Development, Fertility and Family Planning

I confirm that I have read this submission and believe that I have an appropriate level of expertise to confirm that it is of an acceptable scientific standard, however I have significant reservations, as outlined above.

## References

[1] : Reasons for contraceptive nonuse among women having unmet need for contraception in developing countries. *Stud Fam Plann* .2014;45(2) : 10.1111/j.1728-4465.2014.00382.x 151-69 10.1111/j.1728-4465.2014.00382.x 24931073

[2] : Unmet need for contraception: issues and challenges. *Stud Fam Plann* .2014;45(2) : 10.1111/j.1728-4465.2014.00380.x 105-22 10.1111/j.1728-4465.2014.00380.x 24931071

[3] : Revising Unmet Need for Family Planning. *USAID* .2012; Reference source

# Reviewer report: Reviewer response for version 1

**DOI:** 10.21956/gatesopenres.14097.r28692

**Published:** 2020-03-20

**Authors:** Mellisa Withers

**Affiliations:** 1 Institute on Inequalities in Global Health, University of Southern California Keck School of Medicine, Los Angeles, CA, USA

Thank you for the opportunity to review this manuscript, which explores reasons for unmet need for contraception in 10 countries where fertility transitions have not begun or where they have stagnated. Overall, I thought the manuscript was well-written and the study methodology was sound. Further reduction of TFR, especially in PTCs, can have numerous benefits and the results of this study could have important policy implications. As the authors highlighted, using national or population-level data can mask important findings at the individual level. Therefore, I thought this study was useful and has many strengths. I thoroughly enjoyed reading it.

 Here are my specific comments:

1. I appreciated the use of DHS data from 10 countries, half of which have not experienced fertility declines and half of which have stagnated. I also thought the use of the 2 recent DHS surveys to look at changes over time in terms of reasons for non-use of contraception was interesting. I thought separating the countries into PTC and TC was useful because the factors that may influence non-use of contraception can be quite different. And, in fact, this was one of the major findings of this study.
2. The authors did a commendable job in reporting on the existing literature and theoretical frameworks used to explain fertility transitions.
3. The methods were clearly stated and the variables were adequately defined.
4. The study uses DHS data from 10 countries. As we know, the DHS data have many limitations. One of the most significant limitations relevant to this study is that it is difficult to measure fertility intentions using surveys with close-ended response categories. Offering simple response options (yes, no, unsure) does not adequately measure the complexity of this decision. Fertility decisions are often made based on a range of social, economic and contextual factors that may change significantly over time. One major limitation of the data is that it is difficult to assess whether the originally reported fertility intention and the self-reported reasons for non-use of contraception adequately reflected the true range of potential reasons or if they reflected response bias. For example, lack of rapport with the interviewer may have increased social desirability bias. The fact that only one reason was given by the majority of women may be an indication that this bias was present. Differences in survey administration may also be factors. However, the authors did a good job in working with the data that were available in the DHS (with all of its challenges) to overcome some of these issues, or at least acknowledge them.
5. While the authors were clear about how they decided which countries fell into the category of “stagnant”, the TFR in these countries and the background behind the fertility lag is quite diverse. For example, Indonesia experienced dramatic declines in TFR much earlier than many other countries. And as the DR and Indonesia’s TFRs are close to replacement level, I wonder if it would be worth highlighting that further reductions in countries such as this are going to be more challenging than reducing fertility in the countries with higher TFRs. Therefore, further disaggregating the data to identify target populations can help provide insight into where to invest more family planning resources.
6. There are many explanations for lack of contraceptive use which vary greatly in different contexts. This was highlighted in the introduction section. The results highlight that multi-dimensional programs are required to further reduce fertility rates. Clearly more work is needed in PTC with regard to information and education. However, relying on educational campaigns to encourage contraceptive uptake will likely not be successful without addressing other major barriers, such as opposition by male partners or fatalistic attitudes. This part of the analysis was the most interesting to me. In particular, that the results demonstrate the need to consider the impact of partner opposition, which has been cited in numerous studies as a major barrier to contraceptive use, especially in sub-Saharan Africa.
7. I found it quite interesting that, for most countries, the unmet need had not declined between baseline and follow-up. In fact, in half of the countries it had actually increased (Chad, DRC, Mozambique, Cameroon, Egypt). And only Indonesia, Ghana and Namibia had seen declines in unmet need of greater than 1% between baseline and follow-up. As the authors pointed out, the unmet need rates were highest among PTCs at both baseline and follow-up. These rates were the least likely to have changed.  In terms of the results, the CPR did not change at all on average in TCs. And it had actually decreased by 2% on average in PTCs—to a low 15% at endline. This dismal figures show that high rates of unmet need persist and there is more work to be done. I wonder if there were cases of countries that have no change in terms of these variables—such as unmet need for contraception--without not falling into the PTC or TC countries included in this study. Would the reasons for non-use be similar for these countries? And if so, how would this translate into policy recommendations in such countries? Maybe looking at persistent (or increasing) unmet need is even more critical than looking at stagnation in TFR? This was likely beyond the scope of this paper but would be worth exploring.
8. The perceived low fecundability as a barrier to contraceptive use is an interesting finding that is consistent with the literature that shows that a high rate of unintended pregnancy among postpartum women, for example. But it is hard to know if these women are in fact at high risk of pregnancy--was the DHS data for these groups is an accurate reflection (should they truly be counted in the group of unmet need for example?) Or is this another limitation of the data? If it is true, increased attention from family planning programs may be warranted among this group.

Is the work clearly and accurately presented and does it cite the current literature?

Yes

If applicable, is the statistical analysis and its interpretation appropriate?

Yes

Are all the source data underlying the results available to ensure full reproducibility?

Yes

Is the study design appropriate and is the work technically sound?

Yes

Are the conclusions drawn adequately supported by the results?

Yes

Are sufficient details of methods and analysis provided to allow replication by others?

Yes

Reviewer Expertise:

Global sexual and reproductive health, gender-based violence

I confirm that I have read this submission and believe that I have an appropriate level of expertise to confirm that it is of an acceptable scientific standard.

# Reviewer report: Reviewer response for version 1

**DOI:** 10.21956/gatesopenres.14097.r27882

**Published:** 2019-10-08

**Authors:** Thomas Spoorenberg

**Affiliations:** 1 United Nations Population Division, New York City, NY, USA

The article offers an interesting analysis that tries to understand better the reasons behind the stalls in fertility levels in developing countries. Using Demographic and Health Survey (DHS) data, ten countries are selected; five that are in a pre-transitional situation (i.e. where the fertility transition is yet to start) and five other that are in transition but where the fertility decline stalled. The paper focuses on the investigation of the reasons women are not using contraception in these contexts. The analysis shows that the reasons for contraceptive non-use are somewhat distinct in pre-transitional and transitional countries with fertility stalls. Based on the results of the study, implications for program formulation and policy making are drawn.

 While I like the idea of trying to understand better what could contribute to explain the fertility stalls (both when the total fertility rate has not been declining or when the total fertility rate has already started to decline), the article presents some issues that would need to be better addressed.

 It is true that multiple studies have proposed distinct assessment and measurement of fertility stalls with varying level of robustness. Yet, most of these contributions stress the potential bias related to surveys' data quality that constitute the base to those endeavors. In this article, the way the authors have decided to operationalize fertility stalls - i.e. taking two point estimates *n*-years apart - is however too simplistic.

 The countries included in the analysis are selected based on 2 criteria: 1) at least two DHS conducted since 2000 and with final datasets available as of March 2018; 2) countries that have stalled or increasing TFR estimates above replacement level. I would argue that both criteria are problematic.

 While very useful, DHS data present some quality issues. It is well acknowledged that birth information collected in DHS are affected by birth omissions and/or birth displacements that affect the estimation of the fertility levels (among many, see Pullum and Becker, 2014 ^1^; Schoumaker 2014 ^2^). For various reasons, the extent of these biases differs between countries and from one survey to the next. Hull and Hartanto (2009 ^3^) have also shown for Indonesia that the population included in the DHS sample is biased. I would invite the authors to discuss some of these points and how they affect the analysis conducted. The issues of data quality are therefore affecting the identification of fertility stalls. While such issues are much less discussed in studies focusing on contraceptive use, similar biases could also affect the estimation of contraceptive use/non-use. For example, the estimates and projections of family planning indicators 2019 published by the United Nations Population Division ( https://www.un.org/en/development/desa/population/theme/family-planning/cp_model.asp) illustrate some inter-survey variability. It would be good if the authors could discuss in more details in the 'Methods' section some of these biases.

 The reason why I think a more thorough discussion of these issues is important is because some of the variations that are presented and discussed in the paper - variations on which programmatic implications are drawn and policy recommendations are made - could be the product of differences in the data quality from one survey to the next, rather than revealing real changes at work.

 In addition, when presenting the results of the changes in unmet need, contraceptive prevalence, etc., the study relies only on point estimates without providing confidence intervals (CIs). The values presented are drawn from sample surveys, therefore it would be indicated to present them with CIs. To note, CIs are provided for TFR estimates in Table 1. In passing, I would like to bring to the attention of the authors that if the TFR value of the upper limit of the CI are taken in the baseline sample survey and the TFR value of the lower limit of the CI for the endline sample survey, some countries would not be selected as experiencing stalling fertility. I bring this point forward in the perspective of the discussion above and as well as a further illustration of the too simplistic rule used to select the countries with stalling fertility.

 In line with the points made herein I would advise the authors to use the recent article of Schoumaker (2019 ^4^) that offers one of the most exhaustive and authoritative treatments of assessing and estimating fertility stalls. I understand that this article was published after the initial submission of the present paper and could therefore not be included in the text. Yet, it would be good to refer to this new study in a revised version of the text. I would also recommend the authors to discuss in more depth how the several data quality issues affect the outcomes of their analysis.

 A second important point is to what extent the results of the analysis are specific to the countries experiencing stalling fertility. Many other countries recorded similar changes in contraceptive non-use without displaying a stall in fertility. For example, in India, the latest 2015-2016 DHS (NFHS-IV) indicates a decline in contraceptive prevalence and an increase in unmet need but no fertility stall has been recorded in the country. Are the reasons identified for the group of countries with stalling fertility really specific to these countries? Similarly, are the differences in reasons for contraceptive non-use between pre-transitional and transitional countries identified in the study specific to countries experiencing fertility stalls? I expect that the reasons identified in the study are found in other countries where fertility has been continuously falling. In other words, the reasons for contraceptive non-use that the study identified do not explain why fertility has been stalling in the selected countries.

 All in all, the study would benefit of a more thorough discussion of how data quality may affect the results, as well as a more critical assessment of whether the changes in contraceptive non-use, as well as the reasons for not using contraception, are really driving the fertility stalls in the selected countries.

Is the work clearly and accurately presented and does it cite the current literature?

Partly

If applicable, is the statistical analysis and its interpretation appropriate?

Yes

Are all the source data underlying the results available to ensure full reproducibility?

Yes

Is the study design appropriate and is the work technically sound?

No

Are the conclusions drawn adequately supported by the results?

Yes

Are sufficient details of methods and analysis provided to allow replication by others?

Yes

Reviewer Expertise:

Demography, Fertility, Population estimates and projections, Population and development, analysis of deficient demographic data and indirect demographic methods

I confirm that I have read this submission and believe that I have an appropriate level of expertise to confirm that it is of an acceptable scientific standard, however I have significant reservations, as outlined above.

## References

[1] : Evidence of Omission and Displacement in DHS Birth Histories. *DHS Methodological Reports No. 11. Rockville, Maryland, USA: ICF International.* .2014; Reference source

[2] : Quality and Consistency of DHS Fertility Estimates, 1990 to 2012. *DHS Methodological Reports No. 12. Rockville, Maryland, USA: ICF International.* .2014; Reference source

[3] : Resolving contradictions in Indonesian fertility estimates. *Bulletin of Indonesian Economic Studies* .2009;45(1) : 10.1080/00074910902836197 61-71 10.1080/00074910902836197

[4] : Stalls in Fertility Transitions in sub-Saharan Africa: Revisiting the Evidence. *Stud Fam Plann* .2019;50(3) : 10.1111/sifp.12098 257-278 10.1111/sifp.12098 31385318PMC6771655
